# Synthesis and crystal structure of ((*E*)-{2-[(*E*)-(4-hy­droxynaphthalen-1-yl)methyl­idene]hydrazin-1-yl}(methyl­sulfan­yl)methyl­idene)azanium hydrogen sulfate monohydrate

**DOI:** 10.1107/S2056989016013232

**Published:** 2016-08-26

**Authors:** Oussama Nehar, Samira Louhibi, Leila Boukli-Hacene, Thierry Roisnel

**Affiliations:** a Laboratoire de Chimie Inorganique et Environnement, Université de Tlemcen, BP 119, 13000 Tlemcen, Algeria; b Laboratoire de Chimie Inorganique et Environnement, Universite de Tlemcen, BP 119, 13000 Tlemcen, Algeria; c Centre de Diffractometrie X, UMR 6226 CNRS, Unité Sciences Chimiques de Rennes, Université de Rennes I, 263 Avenue du General Leclerc, 35042 Rennes, France

**Keywords:** crystal structure, thio­semicarbazone, synthesis, hydrogen bonding

## Abstract

In the title hydrated mol­ecular salt, C_13_H_14_N_3_S^+^·HSO_4_^−^·H_2_O, the protonation of the azomethine N atom in sulfuric acid medium involves the formation of the bis­ulfate anion. The mol­ecular structure of the cation is obtained from the thiol tautomer of thio­semicarbazone wherein the naphthalene moiety and the conjugation of the bonds contribute to the planarity of the mol­ecular skeleton. In the crystal, the cation, anion and water mol­ecule of crystallization are linked by a series of O—H⋯O and N—H⋯O hydrogen bonds, forming a three-dimensional network. Within this network, there are also C—H⋯π inter­actions present involving symmetry-related naphthalene rings.

## Abstract 2

In the title mol­ecular salt, C_13_H_14_N_3_S^+^·HSO_4_^−^·H_2_O, the protonation of the azomethine N atom in sulfuric acid medium involves the formation of a bis­ulfate anion. The mol­ecular structure of the cation is obtained from the thiol tautomer of thio­semicarbazone wherein the naphthalene moiety and the conjugation of the bonds contribute to the planarity of the mol­ecular skeleton.

## Chemical context   

**Figure 1:**
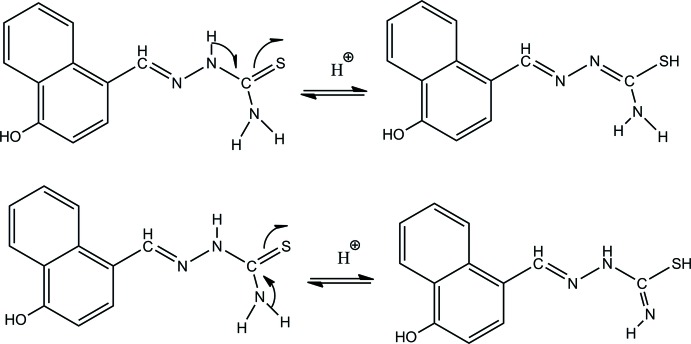
Thio­semicarbazones can exist as *E* and *Z* isomers and they exhibit thione–thiol tautomerism.

Thio­semicarbazones and their metal complexes have been widely explored because of their pharmaceutical properties (Klayman *et al.*, 1983 ▸). These compounds present a wide variety of biological activities, such as anti­tumoral, fungicidal and anti­viral (Tarasconi *et al.*, 2000 ▸), and bactericidal (Abram *et al.*, 1998 ▸). The ability of thio­semicarbazone mol­ecules to chelate with traces of metals in biological systems is believed to be a reason for their activity (Teoh *et al.*, 1999 ▸). The nature of the aldehyde and ketone from which the thio­semicarbazone is obtained and the nature of the substituents attached at the ^+^NH_2_ N atom influence the biological activity (Beraldo & Gambinob, 2004 ▸). Thio­semicarbazones can exist as *E* and *Z* isomers and they exhibit thione–thiol tautomerism, as illus­trated for the title compound in Fig. 1 ▸. Complexation usually takes place *via* dissociation of the acidic proton, resulting in the formation of a five-membered chelate ring (Pal *et al.*, 2002 ▸). The crystal structure of the title compound was determined in order to investigate the extent of electron delocal­ization, the ligand conformation and to explore its biological implications.
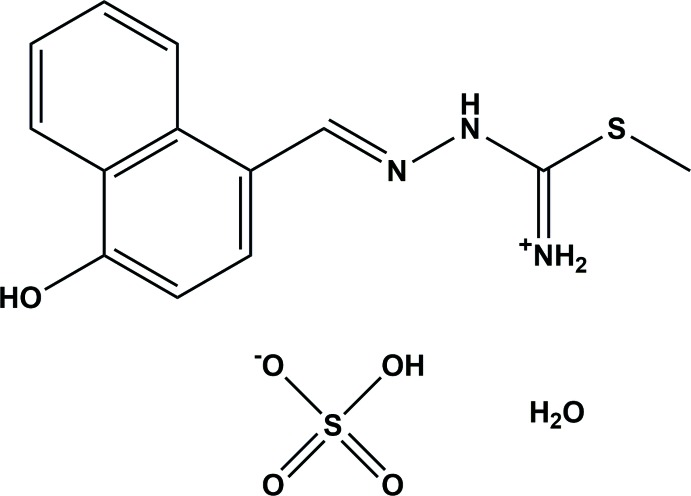


## Structural commentary   

**Figure 2:**
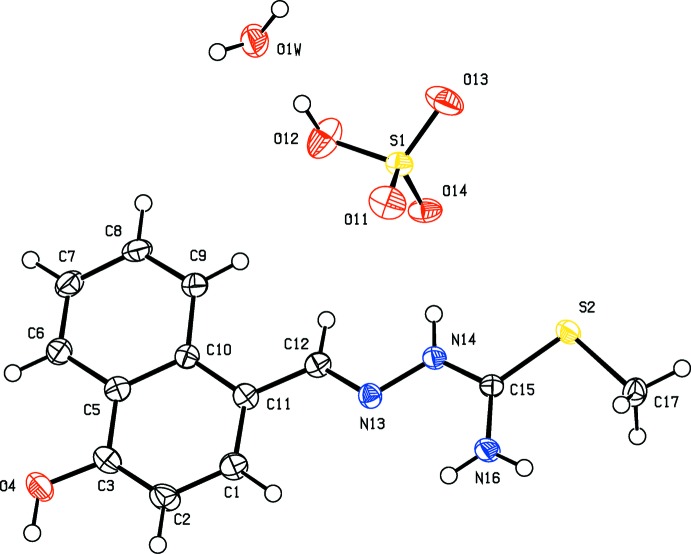
A view of the mol­ecular structure of the title mol­ecular salt, with the atom labelling. Displacement ellipsoids are drawn at the 50% probability level.

The mol­ecular structure of the title mol­ecular salt is illustrated in Fig. 2 ▸. It is composed of three entities: a bis­ulfate anion, a thio­semicarbazone cation and a water mol­ecule of crystallization. The cationic entity shows an *E* conformation with respect to the C12=N13 bond and is approximately planar, the maximum deviation from the mean plane through the 18 non-hydrogen atoms being 0.118 (2) Å for atom C12. This planarity is due to electron delocalization along the cationic entity backbone. Bond lengths and angles are close to those observed for similar (methyl­idene)hydrazinecarbo­thio­amide derivatives (Gangadharan *et al.*, 2015 ▸; Joseph *et al.*, 2004 ▸; Houari *et al.*, 2013 ▸.)

## Supra­molecular features   

**Figure 3:**
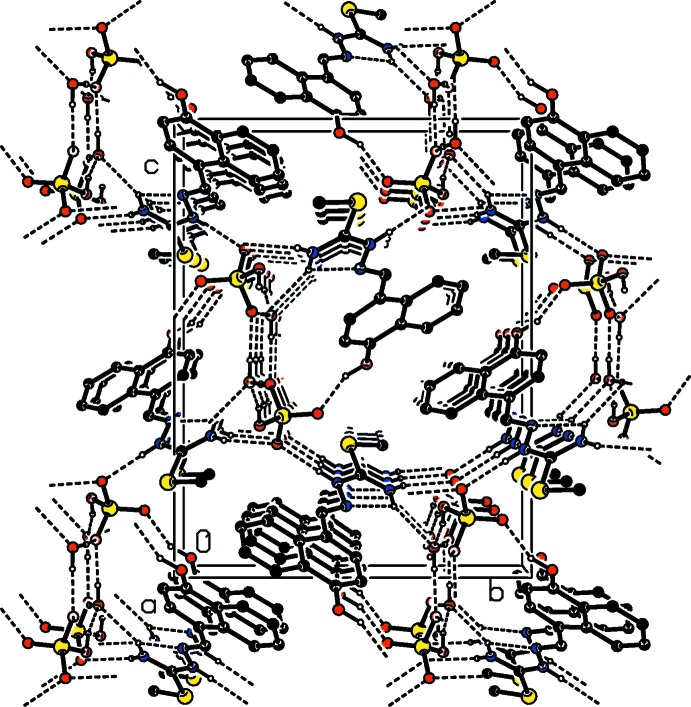
A view along the *a* axis of the crystal packing of the title mol­ecular salt. The hydrogen bonds are drawn as dashed lines (see Table 1 ▸) and the C-bound H atoms have been omitted for clarity.

In the crystal, there is an extensive hydrogen-bonding network present. The cation, anion and water mol­ecule of crystallization are linked by a series of O—H⋯O and N—H⋯O hydrogen bonds, forming a three-dimensional network (Table 1 ▸ and Fig. 3 ▸). Within this network there are also C—H⋯π inter­actions present involving symmetry-related naphthalene rings (Table 1 ▸).

## Database survey   

A search of the Cambridge Structural Database (CSD, Version 5.37, update May 2016; Groom *et al.*, 2016 ▸) for the *S*-methyl (methyl­idene)thio­semicarbazidium cation substructure gave two hits, *viz. S*-methyl-*N*′-(pyrrolyl-2′-methyl­ene)iso­thio­semicarbazidium iodide monohydrate (JIHZUV; Bourosh *et al.*, 1990 ▸) and 8-quinoline­aldehyde *S*-methyl­thio­semicarbazone hydro­chloride dihydrate (RUJXOK; Botoshansky *et al.*, 2009 ▸). Only the coordinates for the latter structure were available. The cation in RUJXOK, is relatively planar and the bond lengths and angles in the *S*-methyl (methyl­idene)thio­semicarbazidium moiety are similar to those observed for the title compound.

## Synthesis and crystallization   

**Figure 4:**
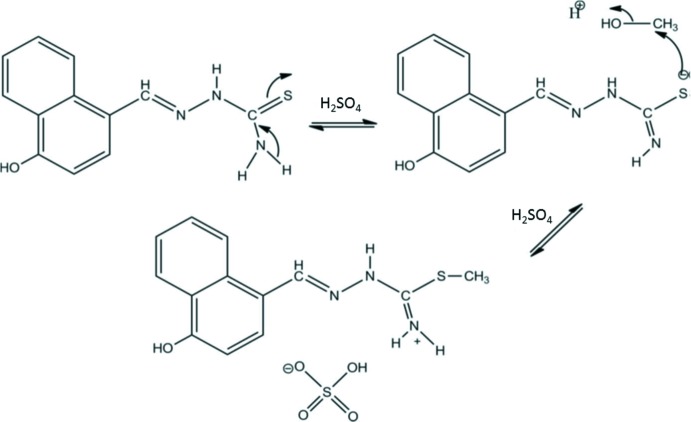
The synthesis of the title mol­ecular salt.

The synthesis of the title mol­ecular salt is illustrated in Fig. 4 ▸. An equimolar amount of thio­semicarbazide 10 mmol (0.91 g) and 3-hy­droxy-2-naphthaldehyde 10 mmol (1.72 g) were dissolved in a mixture of methanol and water (30 ml, 50%) and refluxed for 5 h in the presence of a catalytic amount of glacial sulfuric acid. Brown crystals suitable for X-ray diffraction analysis were obtained after slow evaporation of the solution.

## Refinement   

Crystal data, data collection and structure refinement details are summarized in Table 2 ▸. The hy­droxy H atom was located in a difference Fourier map and freely refined. The water and N-bound H atoms were located in difference Fourier maps and refined with distance restraints O—H = 0.84 (2) Å and N—H = 0.88 (2) Å. The C-bound H atoms were included in calculated positions and treated as riding atoms, with C—H = 0.95–0.98 Å and *U*_iso_(H) = 1.5*U*_eq_(C) for methyl H atoms and 1.2*U*_eq_(C) otherwise.

## Supplementary Material

Crystal structure: contains datablock(s) I, Global. DOI: 10.1107/S2056989016013232/su5320sup1.cif

Structure factors: contains datablock(s) I. DOI: 10.1107/S2056989016013232/su5320Isup2.hkl

Supporting information file. DOI: 10.1107/S2056989016013232/su5320Isup3.cml

CCDC reference: 1451398

Additional supporting information: crystallographic information; 3D view; checkCIF report

## Figures and Tables

**Table 1:** Hydrogen-bond geometry (Å, °) *Cg*1 and *Cg*2 are the centroids of rings C1–C3/C5/C10/C11 and C5–C10, respectively.

*D*—H⋯*A*	*D*—H	H⋯*A*	*D*⋯*A*	*D*—H⋯*A*
O1*W*—H1*WA*⋯O13^i^	0.89 (3)	1.96 (3)	2.791 (4)	155 (5)
O1*W*—H1*WB*⋯O13^ii^	0.86 (2)	1.88 (3)	2.732 (3)	172 (5)
O4—H4*O*⋯O11^iii^	0.96 (6)	1.84 (6)	2.719 (3)	153 (6)
O12—H12*O*⋯O1*W*	0.94 (5)	1.61 (5)	2.543 (4)	168 (5)
N14—H14⋯O14	0.86 (2)	2.00 (2)	2.860 (3)	176 (4)
N16—H16*A*⋯O1*W* ^iv^	0.86 (2)	2.32 (3)	3.046 (4)	142 (4)
N16—H16*B*⋯O14^v^	0.84 (2)	2.20 (3)	2.937 (3)	147 (4)
C6—H6⋯*Cg*2^vi^	0.95	2.67	3.451 (3)	140
C7—H7⋯*Cg*1^vi^	0.95	2.94	3.622 (3)	130

Symmetry codes: (i) 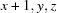; (ii) 
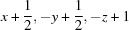
; (iii) 
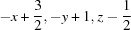
; (iv) 
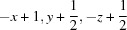
; (v) 
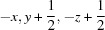
; (vi) 
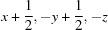
.

**Table 2:** Experimental details

Crystal data	
Chemical formula	C_13_H_14_N_3_OS^+^·HO_4_S^−^·H_2_O
*M*_r_	375.41
Crystal system, space group	Orthorhombic, *P*2_1_2_1_2_1_
Temperature (K)	150
*a*, *b*, *c* (Å)	6.3726 (5), 14.2549 (11), 18.2817 (12)
*V* (Å^3^)	1660.7 (2)
*Z*	4
Radiation type	Mo *K*α
μ (mm^−1^)	0.36
Crystal size (mm)	0.42 × 0.33 × 0.19

Data collection	
Diffractometer	Bruker D8 VENTURE
Absorption correction	Multi-scan (*SADABS*; Bruker, 2015 ▸)
*T*_min_, *T*_max_	0.752, 0.935
No. of measured, independent and observed [*I* > 2σ(*I*)] reflections	19123, 3786, 3586
*R*_int_	0.073
(sin θ/λ)_max_ (Å^−1^)	0.649

Refinement	
*R*[*F* ^2^ > 2σ(*F* ^2^)], *wR*(*F* ^2^), *S*	0.039, 0.103, 1.07
No. of reflections	3786
No. of parameters	247
No. of restraints	5
H-atom treatment	H atoms treated by a mixture of independent and constrained refinement
Δρ_max_, Δρ_min_ (e Å^−3^)	0.36, −0.33
Absolute structure	Flack *x* determined using 1477 quotients [(*I* ^+^)−(*I* ^−^)]/[(*I* ^+^)+(*I* ^−^)] (Parsons *et al.*, 2013 ▸)
Absolute structure parameter	0.03 (5)

Computer programs: *APEX3* and *SAINT* (Bruker, 2015 ▸), *SIR97* (Altomare *et al.*, 1999 ▸), *SHELXL2014* (Sheldrick, 2015 ▸) and *PLATON* (Spek, 2009 ▸).

## supplementary crystallographic information

### Crystal data

**Table:**

C_13_H_14_N_3_OS^+^·HO_4_S^−^·H_2_O	*D*_x_ = 1.501 Mg m^−^^3^
*M**_r_* = 375.41	Mo *K*α radiation, λ = 0.71073 Å
Orthorhombic, *P*2_1_2_1_2_1_	Cell parameters from 9907 reflections
*a* = 6.3726 (5) Å	θ = 2.9–27.5°
*b* = 14.2549 (11) Å	µ = 0.36 mm^−^^1^
*c* = 18.2817 (12) Å	*T* = 150 K
*V* = 1660.7 (2) Å^3^	Block, brown
*Z* = 4	0.42 × 0.33 × 0.19 mm
*F*(000) = 784	

### Data collection

**Table:**

Bruker D8 VENTURE diffractometer	3586 reflections with *I* > 2σ(*I*)
Multilayer monochromator	*R*_int_ = 0.073
rotation images scans	θ_max_ = 27.5°, θ_min_ = 3.4°
Absorption correction: multi-scan (SADABS; Bruker, 2015)	*h* = −8→8
*T*_min_ = 0.752, *T*_max_ = 0.935	*k* = −18→18
19123 measured reflections	*l* = −23→23
3786 independent reflections	

### Refinement

**Table:**

Refinement on *F*^2^	Hydrogen site location: mixed
Least-squares matrix: full	H atoms treated by a mixture of independent and constrained refinement
*R*[*F*^2^ > 2σ(*F*^2^)] = 0.039	*w* = 1/[σ^2^(*F*_o_^2^) + (0.0581*P*)^2^ + 0.4752*P*] where *P* = (*F*_o_^2^ + 2*F*_c_^2^)/3
*wR*(*F*^2^) = 0.103	(Δ/σ)_max_ < 0.001
*S* = 1.07	Δρ_max_ = 0.36 e Å^−^^3^
3786 reflections	Δρ_min_ = −0.33 e Å^−^^3^
247 parameters	Extinction correction: SHELXL2014 (Sheldrick, 2015), Fc^*^=kFc[1+0.001xFc^2^λ^3^/sin(2θ)]^-1/4^
5 restraints	Extinction coefficient: 0.027 (5)
Primary atom site location: structure-invariant direct methods	Absolute structure: Flack *x* determined using 1477 quotients [(*I*^+^)-(*I*^-^)]/[(*I*^+^)+(*I*^-^)] (Parsons *et al.*, 2013)
Secondary atom site location: difference Fourier map	Absolute structure parameter: 0.03 (5)

### Special details

**Table:**

Geometry. All esds (except the esd in the dihedral angle between two l.s. planes) are estimated using the full covariance matrix. The cell esds are taken into account individually in the estimation of esds in distances, angles and torsion angles; correlations between esds in cell parameters are only used when they are defined by crystal symmetry. An approximate (isotropic) treatment of cell esds is used for estimating esds involving l.s. planes.

### Fractional atomic coordinates and isotropic or equivalent isotropic displacement parameters (Å^2^)

**Table:**

	*x*	*y*	*z*	*U*_iso_*/*U*_eq_	
S1	0.33252 (11)	0.30710 (5)	0.35547 (4)	0.0214 (2)	
C1	0.6277 (5)	0.5080 (2)	0.06062 (17)	0.0274 (6)	
H1	0.5211	0.5545	0.0624	0.033*	
C2	0.8011 (5)	0.5215 (2)	0.01485 (18)	0.0302 (7)	
H2	0.8125	0.5775	−0.0130	0.036*	
C3	0.9553 (5)	0.4542 (2)	0.00993 (16)	0.0244 (6)	
O4	1.1258 (4)	0.46247 (17)	−0.03340 (14)	0.0334 (6)	
H4O	1.141 (10)	0.520 (5)	−0.059 (3)	0.09 (2)*	
C5	0.9393 (5)	0.3697 (2)	0.05103 (15)	0.0210 (6)	
C6	1.0925 (5)	0.2980 (2)	0.04354 (16)	0.0256 (6)	
H6	1.2085	0.3069	0.0117	0.031*	
C7	1.0739 (5)	0.2162 (2)	0.08197 (17)	0.0308 (7)	
H7	1.1746	0.1678	0.0755	0.037*	
C8	0.9071 (6)	0.2033 (2)	0.13084 (17)	0.0299 (7)	
H8	0.8977	0.1467	0.1581	0.036*	
C9	0.7567 (5)	0.2716 (2)	0.13989 (16)	0.0246 (6)	
H9	0.6458	0.2620	0.1738	0.029*	
C10	0.7656 (4)	0.3566 (2)	0.09897 (14)	0.0192 (5)	
C11	0.6085 (5)	0.4279 (2)	0.10353 (16)	0.0215 (6)	
C12	0.4268 (5)	0.4151 (2)	0.15100 (15)	0.0220 (6)	
H12A	0.4066	0.3563	0.1744	0.026*	
N13	0.2939 (4)	0.48088 (17)	0.16192 (12)	0.0205 (5)	
N14	0.1329 (4)	0.45700 (16)	0.20919 (13)	0.0202 (5)	
H14	0.144 (7)	0.406 (2)	0.234 (2)	0.040 (11)*	
C15	−0.0120 (4)	0.5212 (2)	0.22410 (14)	0.0182 (5)	
N16	−0.0075 (4)	0.60276 (18)	0.19085 (14)	0.0249 (5)	
H16B	−0.103 (5)	0.641 (2)	0.199 (2)	0.032 (10)*	
H16A	0.084 (5)	0.614 (3)	0.1575 (18)	0.036 (11)*	
C17	−0.3802 (5)	0.5843 (2)	0.28398 (18)	0.0253 (6)	
H17A	−0.4311	0.5911	0.2337	0.038*	
H17B	−0.3101	0.6423	0.2992	0.038*	
H17C	−0.4990	0.5718	0.3166	0.038*	
S2	−0.19735 (11)	0.48825 (5)	0.28856 (4)	0.0245 (2)	
O11	0.3992 (4)	0.40352 (16)	0.36112 (13)	0.0348 (6)	
O12	0.5287 (4)	0.2471 (2)	0.33653 (15)	0.0442 (7)	
H12O	0.631 (8)	0.246 (4)	0.374 (3)	0.059 (14)*	
O13	0.2455 (5)	0.2712 (2)	0.42322 (13)	0.0433 (7)	
O14	0.1937 (4)	0.29015 (14)	0.29348 (11)	0.0278 (5)	
O1W	0.8206 (4)	0.2244 (2)	0.42969 (13)	0.0385 (6)	
H1WA	0.939 (6)	0.256 (4)	0.426 (3)	0.060 (16)*	
H1WB	0.784 (8)	0.225 (3)	0.4750 (15)	0.061 (15)*	

### Atomic displacement parameters (Å^2^)

**Table:**

	*U*^11^	*U*^22^	*U*^33^	*U*^12^	*U*^13^	*U*^23^
S1	0.0240 (3)	0.0184 (3)	0.0220 (3)	−0.0050 (3)	−0.0028 (3)	0.0010 (2)
C1	0.0309 (15)	0.0164 (14)	0.0349 (15)	0.0031 (12)	0.0053 (12)	0.0021 (11)
C2	0.0386 (17)	0.0186 (13)	0.0333 (15)	0.0000 (14)	0.0083 (14)	0.0045 (12)
C3	0.0286 (15)	0.0232 (14)	0.0215 (13)	−0.0062 (11)	0.0019 (12)	−0.0019 (11)
O4	0.0336 (13)	0.0323 (13)	0.0344 (12)	0.0011 (10)	0.0136 (10)	0.0079 (10)
C5	0.0226 (14)	0.0217 (14)	0.0186 (12)	−0.0016 (11)	−0.0032 (11)	−0.0032 (10)
C6	0.0250 (14)	0.0321 (17)	0.0196 (13)	0.0047 (13)	0.0002 (11)	−0.0013 (12)
C7	0.0324 (17)	0.0351 (18)	0.0248 (14)	0.0145 (14)	0.0001 (13)	0.0014 (13)
C8	0.0347 (16)	0.0299 (16)	0.0253 (14)	0.0108 (14)	0.0001 (13)	0.0084 (12)
C9	0.0254 (14)	0.0265 (14)	0.0218 (13)	0.0037 (11)	0.0008 (11)	0.0031 (11)
C10	0.0202 (13)	0.0207 (13)	0.0166 (12)	−0.0004 (10)	−0.0020 (10)	−0.0027 (10)
C11	0.0244 (14)	0.0176 (13)	0.0226 (13)	−0.0011 (11)	0.0024 (11)	−0.0026 (10)
C12	0.0234 (14)	0.0192 (13)	0.0233 (13)	−0.0003 (11)	−0.0012 (11)	−0.0009 (11)
N13	0.0204 (11)	0.0196 (11)	0.0215 (11)	0.0001 (10)	0.0021 (9)	−0.0011 (9)
N14	0.0212 (11)	0.0160 (11)	0.0233 (11)	0.0010 (8)	0.0028 (10)	0.0028 (9)
C15	0.0198 (12)	0.0182 (12)	0.0166 (11)	−0.0002 (10)	−0.0008 (9)	−0.0006 (10)
N16	0.0287 (13)	0.0187 (12)	0.0273 (12)	0.0058 (10)	0.0084 (11)	0.0056 (10)
C17	0.0218 (13)	0.0214 (13)	0.0326 (15)	0.0023 (11)	0.0030 (12)	−0.0007 (12)
S2	0.0264 (4)	0.0209 (3)	0.0263 (3)	0.0027 (3)	0.0080 (3)	0.0056 (3)
O11	0.0456 (14)	0.0215 (11)	0.0372 (12)	−0.0120 (10)	−0.0030 (11)	−0.0060 (10)
O12	0.0395 (14)	0.0493 (17)	0.0437 (14)	0.0146 (13)	−0.0118 (12)	−0.0185 (13)
O13	0.0443 (15)	0.0602 (17)	0.0254 (11)	−0.0204 (13)	−0.0057 (10)	0.0137 (11)
O14	0.0353 (12)	0.0209 (10)	0.0271 (10)	−0.0059 (9)	−0.0089 (10)	0.0040 (8)
O1W	0.0277 (12)	0.0604 (17)	0.0274 (11)	0.0027 (12)	0.0015 (10)	0.0012 (11)

### Geometric parameters (Å, º)

**Table:**

S1—O11	1.442 (2)	C9—C10	1.425 (4)
S1—O13	1.450 (2)	C9—H9	0.9500
S1—O14	1.458 (2)	C10—C11	1.429 (4)
S1—O12	1.554 (3)	C11—C12	1.459 (4)
C1—C11	1.391 (4)	C12—N13	1.279 (4)
C1—C2	1.399 (4)	C12—H12A	0.9500
C1—H1	0.9500	N13—N14	1.384 (3)
C2—C3	1.375 (4)	N14—C15	1.328 (4)
C2—H2	0.9500	N14—H14	0.86 (2)
C3—O4	1.350 (4)	C15—N16	1.313 (4)
C3—C5	1.424 (4)	C15—S2	1.733 (3)
O4—H4O	0.96 (6)	N16—H16B	0.84 (2)
C5—C6	1.420 (4)	N16—H16A	0.86 (2)
C5—C10	1.424 (4)	C17—S2	1.800 (3)
C6—C7	1.366 (5)	C17—H17A	0.9800
C6—H6	0.9500	C17—H17B	0.9800
C7—C8	1.400 (5)	C17—H17C	0.9800
C7—H7	0.9500	O12—H12O	0.94 (5)
C8—C9	1.376 (4)	O1W—H1WA	0.89 (3)
C8—H8	0.9500	O1W—H1WB	0.86 (2)
			
O11—S1—O13	112.83 (16)	C10—C9—H9	119.6
O11—S1—O14	113.10 (14)	C5—C10—C9	117.7 (3)
O13—S1—O14	111.92 (14)	C5—C10—C11	119.2 (3)
O11—S1—O12	107.66 (17)	C9—C10—C11	123.1 (3)
O13—S1—O12	107.67 (18)	C1—C11—C10	119.3 (3)
O14—S1—O12	102.94 (13)	C1—C11—C12	120.5 (3)
C11—C1—C2	121.3 (3)	C10—C11—C12	120.1 (3)
C11—C1—H1	119.4	N13—C12—C11	121.8 (3)
C2—C1—H1	119.4	N13—C12—H12A	119.1
C3—C2—C1	120.5 (3)	C11—C12—H12A	119.1
C3—C2—H2	119.7	C12—N13—N14	114.1 (2)
C1—C2—H2	119.7	C15—N14—N13	118.3 (2)
O4—C3—C2	123.5 (3)	C15—N14—H14	122 (3)
O4—C3—C5	116.2 (3)	N13—N14—H14	118 (3)
C2—C3—C5	120.3 (3)	N16—C15—N14	120.0 (3)
C3—O4—H4O	117 (4)	N16—C15—S2	124.7 (2)
C6—C5—C10	120.0 (3)	N14—C15—S2	115.3 (2)
C6—C5—C3	120.6 (3)	C15—N16—H16B	119 (3)
C10—C5—C3	119.4 (3)	C15—N16—H16A	120 (3)
C7—C6—C5	120.3 (3)	H16B—N16—H16A	120 (4)
C7—C6—H6	119.9	S2—C17—H17A	109.5
C5—C6—H6	119.9	S2—C17—H17B	109.5
C6—C7—C8	120.4 (3)	H17A—C17—H17B	109.5
C6—C7—H7	119.8	S2—C17—H17C	109.5
C8—C7—H7	119.8	H17A—C17—H17C	109.5
C9—C8—C7	120.8 (3)	H17B—C17—H17C	109.5
C9—C8—H8	119.6	C15—S2—C17	101.74 (14)
C7—C8—H8	119.6	S1—O12—H12O	114 (3)
C8—C9—C10	120.7 (3)	H1WA—O1W—H1WB	108 (5)
C8—C9—H9	119.6		
			
C11—C1—C2—C3	1.5 (5)	C8—C9—C10—C5	−2.5 (4)
C1—C2—C3—O4	179.6 (3)	C8—C9—C10—C11	176.9 (3)
C1—C2—C3—C5	0.5 (5)	C2—C1—C11—C10	−2.0 (5)
O4—C3—C5—C6	−2.3 (4)	C2—C1—C11—C12	179.9 (3)
C2—C3—C5—C6	176.9 (3)	C5—C10—C11—C1	0.6 (4)
O4—C3—C5—C10	178.9 (3)	C9—C10—C11—C1	−178.9 (3)
C2—C3—C5—C10	−1.9 (4)	C5—C10—C11—C12	178.6 (3)
C10—C5—C6—C7	0.2 (4)	C9—C10—C11—C12	−0.8 (4)
C3—C5—C6—C7	−178.6 (3)	C1—C11—C12—N13	−8.9 (4)
C5—C6—C7—C8	−2.0 (5)	C10—C11—C12—N13	173.1 (3)
C6—C7—C8—C9	1.5 (5)	C11—C12—N13—N14	−179.2 (2)
C7—C8—C9—C10	0.9 (5)	C12—N13—N14—C15	179.6 (2)
C6—C5—C10—C9	2.0 (4)	N13—N14—C15—N16	4.2 (4)
C3—C5—C10—C9	−179.2 (3)	N13—N14—C15—S2	−175.70 (19)
C6—C5—C10—C11	−177.5 (3)	N16—C15—S2—C17	7.7 (3)
C3—C5—C10—C11	1.4 (4)	N14—C15—S2—C17	−172.4 (2)

### Hydrogen-bond geometry (Å, º)

Cg1 and Cg2 are the centroids of rings C1–C3/C5/C10/C11 and C5–C10, respectively.

**Table:**

*D*—H···*A*	*D*—H	H···*A*	*D*···*A*	*D*—H···*A*
O1*W*—H1*WA*···O13^i^	0.89 (3)	1.96 (3)	2.791 (4)	155 (5)
O1*W*—H1*WB*···O13^ii^	0.86 (2)	1.88 (3)	2.732 (3)	172 (5)
O4—H4*O*···O11^iii^	0.96 (6)	1.84 (6)	2.719 (3)	153 (6)
O12—H12*O*···O1*W*	0.94 (5)	1.61 (5)	2.543 (4)	168 (5)
N14—H14···O14	0.86 (2)	2.00 (2)	2.860 (3)	176 (4)
N16—H16*A*···O1*W*^iv^	0.86 (2)	2.32 (3)	3.046 (4)	142 (4)
N16—H16*B*···O14^v^	0.84 (2)	2.20 (3)	2.937 (3)	147 (4)
C6—H6···*Cg*2^vi^	0.95	2.67	3.451 (3)	140
C7—H7···*Cg*1^vi^	0.95	2.94	3.622 (3)	130

Symmetry codes: (i) *x*+1, *y*, *z*; (ii) *x*+1/2, −*y*+1/2, −*z*+1; (iii) −*x*+3/2, −*y*+1, *z*−1/2; (iv) −*x*+1, *y*+1/2, −*z*+1/2; (v) −*x*, *y*+1/2, −*z*+1/2; (vi) *x*+1/2, −*y*+1/2, −*z*.
